# Dental Treatment Needs in Vancouver Inner-City Elementary School-Aged Children

**DOI:** 10.1155/2013/602791

**Published:** 2013-06-05

**Authors:** F. Samim, J. Aleksejuniene, C. Zed, N. Salimi, C. P. Emperumal

**Affiliations:** ^1^Faculty of Dentistry, University of British Columbia, 2199 Wesbrook Mall, Vancouver, BC, Canada V6T 1Z3; ^2^Department of Oral Health Sciences, Faculty of Dentistry, University of British Columbia, 2199 Wesbrook Mall, Vancouver, BC, Canada V6T 1Z3; ^3^Tamil Nadu Dr. MGR Medical University, Chennai, India

## Abstract

*Aims*. To examine the dental treatment needs of inner-city Vancouver elementary school-aged children and relate them to sociodemographic characteristics. *Methods*. A census sampling comprising 562 children from six out of eight eligible schools was chosen (response rate was 65.4%). Dental treatment needs were assessed based on criteria from the World Health Organization. *Results*. Every third child examined needed at least one restorative treatment. A higher proportion of children born outside Canada were in need of more extensive dental treatments such as pulp care and extractions compared to the children born in Canada. There were no statistically significant differences in dental treatment needs between age, gender, or income groups or between children with or without dental insurance (Chi Squared *P* > 0.05). The best significant predictors (Linear Multiple Regression, *P* > 0.05) of higher dental treatment needs were being born outside Canada, gender, time of last dental visit, and family income. Having dental insurance did not associate with needing less treatment. Conclusion. A high level of unmet dental treatment needs (32%) was found in inner-city Vancouver elementary school-aged children. Children born outside Canada, particularly the ones who recently arrived to Canada, needed more extensive dental treatments than children born in Canada.

## 1. Introduction

Dental caries has a negative impact on an individual's well-being and quality of life [1]. It is considered one of the most common oral health burdens with 60–90% of schoolchildren worldwide experiencing dental caries [2, 3]. Poor dental health is most common in vulnerable populations such as low socioeconomic status families, ethnic minorities, and immigrants [4, 5]. According to data from the 2011 census, in British Columbia (BC) over 80% of the population's increase between 2006 and 2011 was due to immigration [6]. Of all immigrants in BC, 85% chose Vancouver as their place of residence [6], and 88% of BC's new immigrant children during that time period were under 15 years of age [7]. Possibly, these new immigrants face multiple challenges with poor dental health being one of them.

Dental caries is one of the four most expensive diseases to treat [8]. Financial barriers to dental care have been reported by low-income Canadian adults [9]; those who are insured are more likely to visit a dentist compared to those who must pay for their dental care out of pocket [10]. This unmet health care need is common among Canadian children [11]. Moreover, poor children are more likely than their better-off peers to be exposed to different health risks, and these inequities are further increased by barriers to accessing professional dental care [12]. British Columbia has the highest child poverty rate among all Canadian provinces [13]. 

Vancouver inner-city schools are found in areas with the lowest residential stability, highest housing density, and highest population diversity in terms of languages spoken and countries of birth [6]. In Vancouver, due to limited public expenditure, professional dental screenings are provided only for kindergarten children. Thus, the extent of the dental treatment needs of older inner-city Vancouver children is unknown. 

The aim of the present study was to examine dental treatment needs of a cohort of inner-city Vancouver elementary school-aged children and relate these treatment needs to a number of sociodemographic factors.

## 2. Materials and Methods

The study was approved by the University of British Columbia Ethics Board (H11-00621) and by the Vancouver School Board. 

According to the socio-economic mapping provided by the Human Early Learning Partnership, Vancouver School District 39 (Vancouver School Board, 2011) encompasses one of the most disadvantaged areas in British Columbia, Canada [14]. Therefore, the present study focused on inner-city elementary schools found in this school district. Three out of these 11 schools were not eligible for the present study as children from these schools were already receiving dental screenings and services. Thus, a total of eight schools were eligible. Principals of these schools were contacted and informed about the study, and subsequently six out of the eight invited schools consented to participate. 

A census sampling was chosen to obtain an accurate overview of the dental treatment needs of all inner-city Vancouver elementary school-aged children, including the children from kindergarten to grade seven who at the time of the study attended the preselected schools. The recruitment of children was facilitated by school staff and by youth or social workers. A letter of initial contact for parents including a brief summary of the project was translated into the five most common languages according to the demographic profile of inner-city Vancouver. These documents were sent to the children's parents or guardians along with a consent form. 

The study protocol had been previously tested in a sample of 20 children from an inner-city elementary school not included in the eight schools selected for the present study. During the pilot study, feedback from the children and staff was sought. Subsequently, some revisions to the study's protocol were made. The data of the present study was collected from May 2011 to January 2012. A total of three attempts, including follow-up calls, were made to reach the parents and guardians of the children. The final response rate was 65.4%, and the final study sample included 562 children. Information about these children was collected by means of interview, structured questionnaire, and clinical examination. The sociodemographic data of each family's income, education, and dental insurance status was obtained from the Vancouver School Board. The study variables are presented in Table 1.

The clinical examination was done by a trained dentist who was assisted by another experienced dentist and included assessments of dental treatment needs based on the WHO criteria for Oral Health Surveys [15]. The intraexaminer reliability of clinical recordings employing the Cohen's Kappa was tested in a random sample of 20 children. The Kappa test of 0.90 was considered a satisfactory level of intra-examiner reliability. After the clinical examination, a referral letter including the addresses of public dental clinics was provided to the parents or guardians of children who needed operative treatments.

All analyses were performed employing SPSS 21.00 software. Univariate analyses were used to describe the children in terms of a number of socio-demographic factors and to test the data for normalcy in order to prepare for subsequent inferential bivariate and multivariate analyses. Bivariate analyses were employed to test associations between dental treatment needs and socio-demographic factors (Chi Squared test/Fisher's exact test). Linear multiple regression was used to study dental treatment need related outcomes (number of decayed teeth including teeth in need for pulpal care or extractions and total treatments needed) in relation to socio-demographic factors. 

## 3. Results 

The present study comprised a total of 562 children from kindergarten to grade seven, of which 56% were boys and 44% were girls. The sample distribution according to socio-demographic variables is presented in Table 2. More than half of the children (55%) did not have dental insurance, and most were Filipino (20%) or South Asian descent (17%). Of all, 18% of children were not able to answer the question regarding their birthplace, and 19% of children who responded to this question reported that they were born outside Canada. Over one third of examined children had never visited a dentist or did not remember the date of their last dental visit.

Table 3 shows the dental treatment needs and indicates proportions of children who needed specific treatment modalities. Overall, every third examined child (32%) needed to be referred for an operative dental treatment, and similar proportions of children among different age groups were in need of these treatments. A small proportion of children needed extractions of deciduous teeth due to extensive caries, but there were no children in need of extractions of permanent teeth. Most of the children needed one or two surface fillings, and around 10% or more of the younger children (kindergarten, grades 1-2) needed extensive treatments such as four-surface fillings or pulp care in their deciduous dentitions.

Table 4 presents the results of bivariate analyses. There were no statistically significant differences in dental treatment needs between the two genders and between income groups or between children with or without dental insurance. There were statistically significant differences regarding the country of birth, with children born outside of Canada having a higher need for dental treatment as compared to their Canadian-born counterparts. Among the ones who needed treatment, the proportion of the recent immigrants, who had come to Canada within the last two years, was higher (*P* = 0.024) when compared to the ones who had lived in Canada for a longer period of time.

Table 5 presents the results of multivariate testing of the dental treatment needs: the number of decayed teeth and the number of total treatments needed including treatments of teeth which required pulp care and extractions. For both regression models the following predictors were evaluated: gender, age, time passed since last dental visit, having dental insurance, family's education, and family's income. The overall linear regression model for the outcome “number of decayed teeth” was highly statistically significant (*P* < 0.001), and the best statistically significant predictors were gender, born outside Canada, and being from a family with a higher income. These predictors jointly explained 17% of the variation (adjusted *R* square = 0.169). The second multiple regression model for the “number of treatments needed” was also highly statistically significant (*P* < 0.001), and the best predictors were gender (standardized coefficient = 0.300, *P* = 0.010) and being born outside Canada (standardized coefficient = 0.279, *P* = 0.03). These two predictors jointly explained 22% of the variation in the total number of treatments needed (adjusted *R* square = 0.220).

## 4. Discussion 

The present study focused on the overall dental treatment needs and the specific treatment modalities of unmet dental treatment needs of children from Vancouver's inner-city elementary schools. 

Prior to discussing the present study's results, it is important to acknowledge that only simple school-based dental examinations were employed, which may have led to an underestimation of the true extent of the dental treatment needs of this cohort of inner-city elementary school-aged children. 

Overall, a relatively high level of treatment needs was found, as every third child needed to be referred for dental treatments. This high proportion (32%) of Vancouver elementary school-aged children in need of operative treatments is higher than the record of 20% for Canadian population in need of dental treatments as reported in the National Canadian 2007–2009 Survey [16]. Unmet treatment needs in vulnerable children have an impact on children's overall health and wellbeing; thus they should be considered a public health burden [17]. Being born outside Canada was found to be one of the best predictors of higher treatment needs when controlled for having dental insurance and the family's socio-economic status (income and education). When length of residence in Canada was taken into account, we found a significant association between the time since immigration and dental health. More specifically, our study showed that 42% of children who had arrived in Canada within the last two years required restorative dental care compared to only 25% of those who lived in Canada longer. In addition, new immigrants made fewer visits to a dentist despite their higher dental treatment needs. Similarly to our study, recent immigrants to Ontario had used dental services less than immigrants who had been in Canada for six years or more [18]. 

Recent immigrants to Canada are known to be at high risk of poverty, and it has been reported that this risk has significantly increased in recent years [6]. The primary caregivers of Canadian immigrant children often work multiple jobs [19]. Possibly, the burden of untreated dental disease and barriers to accessing professional dental care adds to the everyday challenges of these families. 

In a previous Canadian study, the strongest predictor for higher levels of untreated caries in immigrant children was a lack of dental insurance [20]. However, the present study did not find differences between those with or without dental insurance as both groups had a relatively high level of unmet dental treatment needs. Possibly, multiple factors play a role in the development of unmet treatment needs. Disparities in the oral health status of Canadians have been related to the structure of professional dental care [21, 22] and to multiple barriers such as limited health care availability, language barriers, unequal distribution of dental care providers, lack of available government funded programs, as well as unawareness of low income families about the possibilities for acquiring access to professional dental care for themselves or their children [23, 24]. Similarly, a number of predisposing, enabling, and need-based factors such as language barriers, low socio-economic neighborhoods, lack of dental awareness and education, cultural differences, and immigrant status have been identified as barriers for those seeking access to professional care [25, 26]. 

To address the problem of the relatively high level of unmet treatment needs observed in Vancouver inner-city elementary school-aged children, multiple strategies should be considered. For example, the newly formed Platform for Better Oral Health in Europe is working at a macrolevel by bringing interested associations, groups, and individuals together to alert national governments to oral health problems and to promote policies reducing disparities in oral health [27]. Another strategy for reducing inequalities in dental health may be through health promoting schools based on the WHO's Global School Health Initiative, which was designed to improve the health of children, school personnel, families, and other members of the community [28]. A Canadian example of a school-based oral health promotion program “Brighter Smiles” has been demonstrated as a successful strategy in improving children's oral health [29, 30]. Strategies that target parents and not just children may also help to address oral health disparities in children as it has been reported that parental dental care behaviors have an important effect on the health-focused behaviors of their children [31]. 

## 5. Conclusions 

The unmet dental treatment needs were high in this cohort of Vancouver inner-city elementary school-aged children, and every third child needed an operative dental treatment. Higher treatment needs were found in children who had not visited a dentist within the last year and who had recently immigrated to Canada.

## Figures and Tables

**Table 1:** Operationalization of the study variables.

	Operationalization (codes)
Sociodemographic variables	
Age groups	(0) Kindergarten, (1) grades 1-2,
Gender	(1) Boy, (2) girl.
Ethnicity	(2) Aboriginal, (2) Chinese, (3) European, (4) Filipino, Vietnamese (5), Hindi (6), and other (7).
Family's income	(1) Lowest income group: (from no income to less than 20.000) (2) Medium income group: (20.000–40.000) (3) Highest income group (>40.000)
Born in Canada	(0) Born outside of Canada, (1) in Canada
Having a dental insurance	(1) Yes, no (0)
Last dental visit	(0) Do not remember or never, (1) more than a year ago, (2) within last year.

Treatment needs^#^	
No treatment	(0) A crown is sound or a tooth does not need any professional treatment
One-surface filling*	(1) A tooth needs a one-surface filling.
Two-surface filling*	(2) A tooth needs a two-surface filling.
Three-surface filling*	(3) A tooth needs a three-surface filling.
Four-surface filling	(4) A tooth needs a four-surface filling or a crown.
Pulp care (4)	(5) A tooth needs pulp care prior to restoration with a filling or crown because of deep and extensive caries or because of tooth mutilation or trauma.
Extraction (5)	(6) A tooth needs extraction due to extensive caries, or caries has destroyed a tooth such that it cannot be restored.

^#^Treatment needs recorded based on the WHO criteria.

*One of the codes 1, 2, or 3 was also used to indicate the treatment required to treat primary or secondary caries or to replace an unsatisfactory filling. A filling was considered unsatisfactory if one or more of the following conditions existed: a deficient margin, an overhanging margin, or a fracture of an existing restoration that either caused the filling to loosen or permitted leakage into the dentin and thus discoloration.

**Table 2:** Sample distribution according to sociodemographic variables.

Variables *N* ^#^	Number (%)
Age groups (*N* = 547)	
Kindergarten	90 (16.4)
Grades 1-2	170 (31.0)
Grades 3-4	130 (23.7)
Grades 5–7	159 (29.0)
Gender (*N* = 554)	
Boys	312 (56.3)
Girls	242 (43.7)
Family's income (*N* = 529)	
<20.000	202 (38.2)
20.000–40.000	161 (30.4)
>40.000	166 (31.4)
Having insurance	
No	289 (54.6)
Yes	240 (45.4)
Born in Canada (*N* = 461)	
No	104 (18.5)
Yes	357 (63.5)
Ethnicity (*N* = 559)	
Aboriginal	43 (7.7)
Chinese	59 (10.5)
European	85 (15.1)
Filipino	112 (19.9)
Vietnamese	67 (11.9)
South Asian	98 (17.4)
Other	95 (16.9)
Last dental visit (*N* = 443)	
Within last year	256 (57.8)
More than a year ago	23 (5.2)
Do not remember or never	164 (37.0)
Total children	**562 (100%)**

^#^Missing data due to children's inability to answer, or information was incomplete.

**Table 3:** Dental treatment needs in different age groups (primary and permanent dentitions combined)^#^.

Age groups (*N*)	One-surface filling *N* (%)	Two-surface filling *N* (%)	Three-surface filling *N* (%)	Four-surface filling* *N* (%)	Pulp care *N* (%)	Extraction *N* (% )^#^	Needs treatment *N* (%)
Total (547)	84 (15.4)	81 (14.8)	31 (5.7)	46 (8.3)	55 (10.1)	24 (4.4)	176 (32.1)

Kindergarten (90)	13 (14.4)	15 (16.7)	7 (7.8)	10 (8.8)	12 (13.3)	3 (3.3)	28 (31.1)
Grades 1-2 (169)	19 (11.2)	30 (17.8)	13 (7.7)	19 (11.2)	25 (14.8)	6 (3.6)	53 (31.3)
Grades 3-4 (129)	19 (14.7)	18 (14.0)	6 (4.7)	12 (9.3)	13 (10.1)	8 (6.2)	45 (34.8)
Grades 5–7 (159)	33 (20.8)	18 (11.3)	5 (3.1)	5 (3.1)	5 (3.1)	7 (4.4)	50 (31.4)

Chi-Square Test	*P* = 0.056	*P* = 0.118	*P* = 0.052	*P* = 0.024	*P* = 0.001	*P* = 0.514	*P* = 0.960

^#^Missing data due to children's inability to answer, or information was incomplete.

*Needs a four-surface filling or a crown; ^#^need for extractions in severely damaged teeth; none of the permanent teeth needed to be extracted.

**Table 4:** Dental treatment needs with regard to sociodemographic factors^#^.

	Dental treatment needs (deciduous and permanent dentitions combined)						
	One-surface filling	Two-surface filling	Three-surface filling	Four-surface filling or crown	Pulp care	Extraction	Needs any treatment
	*N* (%)	*N* (%)	*N* (%)	*N* (%)	*N* (%)	*N* (%)	*N* (%)
Gender (*N* = 554)							
Boys	46 (14.9)	46 (14.9)	14 (4.4)	25 (8.0)	31 (10.0)	16 (5.2)	75 (24.3)
Girls	39 (16.1)	36 (14.9)	18 (7.4)	20 (8.2)	24 (9.9)	8 (3.3)	61 (25.2)

	P = 0.390	P = 0.548	P = 0.103	P = 0.554	P = 0.541	P = 0.303	P = 0.438

Income (*N* = 529)							
<20.000	32 (16.0)	34 (17.0)	9 (4.5)	17 (8.5)	23 (11.5)	10 (5.0)	53 (26.5)
20.000–40.000	20 (12.4)	22 (13.7)	8 (5.0)	12 (7.4)	14 (8.7)	6 (3.7)	33 (20.5)
>40.000	29 (17.8)	20 (12.3)	11 (6.7)	14 (8.5)	15 (9.2)	7 (4.3)	44 (27.0)

Chi-Square	*P* = 0.395	*P* = 0.416	*P* = 0.618	*P* = 0.827	*P* = 0.631	*P* = 0.840	*P* = 0.983

Insurance (*N* = 529)							
No	42 (14.5)	45 (15.6)	13 (4.5)	22 (7.6)	28 (9.7)	14 (4.8)	69 (24.0)
Yes	37 (15.6)	31 (13.1)	15 (6.4)	21 (8.7)	23 (9.7)	10 (4.2)	59 (25.0)

	*P* = 0.410	*P* = 0.456	*P* = 0.436	*P* = 0.636	*P* = 1.000	*P* = 0.835	*P* = 0.430

Born in Canada (*N* = 461)							
No	20 (19.2)	24 (23.0)	9 (8.6)	11 (10.5)	16 (15.3)	11 (10.5)	35 (33.7)
Yes	59 (16.6)	49 (13.8)	19 (5.3)	26 (7.2)	30 (8.4)	10 (2.8)	88 (24.8)

	*P* = 0.556	*P* = 0.032	*P* = 0.243	*P* = 0.305	*P* = 0.034	*P* = 0.002	*P* = 0.256

Ethnicity (*N* = 559)							
Aboriginal	5 (12.9)	3 (7.3)	3 (7.3)	3 (7.3)	3 (7.3)	2 (4.8)	7 (17.1)
Chinese	8 (13.7)	7 (12.0)	3 (5.1)	4 (6.7)	6 (10.3)	1 (1.7)	12 (20.7)
European	10 (11.7)	16 (18.8)	12 (14.1)	14 (16.4)	16 (18.8)	3 (3.5)	20 (23.5)
Filipino	15 (13.5)	20 (18.0)	6 (5.4)	11 (9.8)	11 (9.8)	8 (7.2)	30 (27.0)
Vietnamese	7 (10.4)	9 (13.4)	3 (4.4)	4 (5.9)	4 (5.9)	3 (4.4)	15 (22.4)
South Asian	17 (17.3)	11 (11.2)	1 (1.0)	5 (5.1)	8 (8.1)	0 (0.0)	23 (32.6)
Other	24 (25.2)	17 (17.8)	4 (4.2)	5 (5.2)	8 (8.4)	7 (7.3)	31 (32.6)

	*P* = 0.117	*P* = 0.432	*P* = 0.015	*P* = 0.129	*P* = 0.150	*P* = 0.129	*P* = 0.078

Dental Visit (*N* = 443)							
Last year	42 (16.7)	37 (14.7)	15 (6.0)	21 (8.2)	24 (9.5)	11 (4.4)	63 (25.0)
More than 1 year	5 (22.7)	7 (31.8)	2 (9.1)	0 (0.0)	1 (4.5)	2 (9.1)	9 (40.9)
Never/don't know	27 (16.7)	26 (16.0)	12 (7.4)	19 (11.6)	24 (14.8)	6 (3.7)	45 (27.8)

	*P* = 0.762	*P* = 0.110	*P* = 0.547	*P* = 0.150	*P* = 0.108	*P* = 0.791	*P* = 0.256

^#^Chi-Square/Fisher's exact test. Missing data due to children's inability to answer, or information was incomplete.

**Table 5:** Dental treatment needs and sociodemographic factors—linear multiple regression analysis

Dependent outcome: total number of treatments needed Predictors tested: gender, age, dental visit, dental insurance, family's education and income.		
Best predictors	Standardized beta coefficient	*P* values

Gender	0.416	<0.001
Born outside Canada	0.330	<0.001
Last dental visit	0.105	0.092
Family income	0.258	0.015

Model summary: Adjusted *R* square = 0.220, *P* < 0.001		

Dependent outcome: total number of decayed teeth Predictors tested: gender, age, dental visit, dental insurance, family's education and income.		

Best predictors	Standardized beta coefficient	*P* values

Gender	0.300	0.010
Born outside Canada	0.279	0.003

Model summary: Adjusted *R* square = 0.169, *P* < 0.001		
